# Narrative discourse comprehension in Greek-speaking children with autism spectrum disorder: evidence from Aesop’s fables

**DOI:** 10.3389/fpsyg.2026.1877679

**Published:** 2026-09-24

**Authors:** Eleni Peristeri, Maria Kosma, Evangelia Panagiotidou, Evdokia Tagkouli, Maria Andreou

**Affiliations:** 1 Department of Theoretical and Applied Linguistics, School of English, Aristotle University of Thessaloniki, Thessaloniki, Greece; 2 “Aghia Sophia” Children’s Hospital, Athens, Greece; 3 Department of Speech and Language Therapy, University of Peloponnese, Kalamata, Greece

**Keywords:** Aesop’s fables, Autism Spectrum Disorders, language complexity, moral judgment, theory of mind

## Abstract

**Introduction:**

Theory of Mind, i.e., the ability to attribute mental states to others, is critical for social communication but is often impaired in autism. Although narrative tasks have been extensively used to study Theory of Mind in autism, moral judgement of the story protagonists’ actions has not received equal attention. The current study aimed to assess cognitive and affective Theory of Mind, as well as moral reasoning skills in autistic children through Aesop’s fable comprehension.

**Methods:**

In this study, 25 autistic children and two equally-sized groups of typically-developing age- and language-matched children listened to two fables, and answered questions assessing the attribution of affective and cognitive mental states to fable protagonists, as well as their judgment about the moral of each fable. Crucially, the two fables differed in terms of language complexity, which was operationalized in terms of vocabulary and syntax, as well as in the use of decontextualized language, i.e., expressions that anchor events in the past.

**Results:**

We found that the autistic children showed significantly lower comprehension performance than typically-developing age-matched children in affective and cognitive Theory of Mind questions, while no differences were found between autistic and typically-developing language-matched children. Also, the autistic children exhibited considerable weaknesses in verbalizing the moral, however, this weakness depended on the language complexity of the fables.

**Discussion:**

The findings demonstrate that autistic children face challenges in both Theory of Mind and moral judgment in narrative comprehension, particularly when the stimuli are linguistically demanding. Aesop’s fables seem to offer a promising venue as a window into the moral judgment skills of autistic children.

## 1 Introduction

Successful social communication relies on one’s ability to navigate multiple perspectives of the same situations as a function of the individual’s social cognitive skills. The task of ‘reading’ other people’s mind is far from trivial, since perspective-taking covers an expansive continuum of relations across diverse situations and individuals, as well as myriad outcomes, such as cognitive understanding, attribution of emotional states, and moral judgment. The importance of the mechanism that allows for these outcomes, known as Theory of Mind (ToM), has been efficiently illustrated by cases in which ToM is impaired, as in Autism Spectrum Disorder (ASD; Andreou and Skrimpa, 2020; Baron-Cohen, 2000; Kimhi, 2014; Tager-Flusberg, 2007; Peristeri et al., 2023).

Research in ToM in ASD has traditionally used narratives as a press for mental state attribution with a focus on the mindset of the narratives’ story characters. One of the ways in which narratives can represent the inner worlds of characters is through the use of viewpoint markers, i.e., lexical elements that signal that a part of the narrative has to be constructed from the subjective viewpoint of a character (van Krieken et al., 2017). Being an effective narrator highly depends on the individual’s alignment with the perspective of one or more of the story characters as well as their spatio-temporal environments, i.e., the tendencies arising from the sum of the characters’ sensory perceptions, thoughts, emotions, and related processes (Williams, 2021). This alignment may result from lexical viewpoint markers, i.e., inferences about the story characters’ emotional states and feelings, also known as affective ToM terms (e.g., *angry, upset, happy*), or characters’ thoughts and beliefs, also called cognitive ToM terms (i.e., *I think, I believe*; Baldimtsi et al., 2021; Peristeri et al., 2026a; Shah et al., 2019; Shamay-Tsoory et al., 2005; Tager-Flusberg and Sullivan, 2000). The specific studies have highlighted impairment in both ToM aspects, with cognitive ToM being disproportionally more impaired as compared to affective ToM in ASD. Given the prominence of language impairment in ASD, a number of studies has highlighted strong relationships between autistic children’s language skills and their understanding of the story protagonists’ thoughts and feelings, further implying that the use of viewpoint markers is proportional to the language complexity of the produced narrative, mainly in terms of vocabulary and syntax (Andreou et al., 2026a; Peristeri et al., 2017; Peristeri and Andreou, 2025; Westerveld and Roberts, 2017). Difficulty with affective ToM terms has also been associated with autistic children’s impairment in perception and production of vocal emotions (Paul et al., 2005; Peppé et al., 2007; Peristeri et al., 2026). These studies support the importance of structural language skills for narrative comprehension in ASD. However, mental states are often communicated implicitly in a narrative, i.e., they cannot be deduced solely through the vocabulary or/and the morphosyntax of the narrative input, but need to be inferred through one’s ToM abilities.

Various narrative tools have so far been employed to investigate mental state attribution skills (i.e., either cognitive or affective states) in autistic as compared to typically-developing (TD) children. For instance, autistic children’s narrative comprehension and production has been frequently assessed through the picture book ‘Tuesday’ of the Autism Diagnostic Observation Schedule (ADOS; Lord et al., 1999; Banney et al., 2014; Kenan et al., 2019; Kuijper et al., 2017; Rumpf et al., 2012). Additional narrative tools that have been used to evaluate ToM in ASD (Gabig, 2008; Losh and Gordon, 2014; Mäkinen, 2015) include the ‘Frog on His Own’ (Mayer, 1973) and the ‘Bus Story Test’ (Renfrew, 1997a) storybooks. These studies offer converging evidence that the processing of emotional and cognitive viewpoints, such as internal responses and evaluative reactions, is more difficult for autistic as compared to TD children.

One consideration that current narrative designs have not addressed so far in autistic children is their moral thinking about the story characters’ actions or mental states. It is often the case that mental state attribution in real life situations cannot be realized independently from harm detection in the intentions of an agent or the outcomes of their action. Previous research in typical development (Cushman et al., 2013; Jambon and Smetana, 2014; Nunez and Harris, 1998; Yuill and Perner, 1988; Vaish et al., 2010) has shown that children’s ToM skills are affected by the evaluation of the intentions of actors as early as 4 years old, and that the two cognitive processes, i.e., moral judgment and ToM, dynamically interact in shaping TD children’s efficient responsiveness to social situations. In fact, children’s belief attribution skills have been found to be strongly related to their ability to integrate intention and motive information in morally relevant situations as early as the fifth year of age (Baird and Astington, 2004; Dunn et al., 2000).

Research in the links between moral judgment and ToM in ASD is far from clear. In fact, findings tend to show divergences between the two systems that raise important questions for the ToM-moral judgment interdependence view that seems to hold for TD child populations. Fadda et al. (2016) showed that autistic children who scored lower than their TD peers in a second-order false belief ToM task were still able to detect the immoral intention behind a protagonist’s action; however, the autistic children that completely failed in the ToM task tended to show more rigid, negative moral judgments than their autistic peers with higher ToM performance toward actions that broke the rules of morally appropriate behavior. This pattern highlights that advanced ToM skills are not essential for moral judgment in ASD, however, they may account for different dimensions (social transgression detection and emotional arousal to moral transgression) of children’s moral judgment. Interestingly, Li et al. (2014) also found that autistic children had more rigid criteria for what constitutes a moral transgression than their TD peers when cooperating with other individuals, which implies that the way they integrate their moral judgments into their social behavior and decision-making is different from TD children (also see Dempsey et al., 2020a for a review of moral judgment in ASD). Moran et al. (2011) study has also offered evidence in favor of a dissociation between ToM and moral judgment by showing that autistic children were not able to judge accidental and attempted harms as being morally different, despite their preserved performance in a false belief attribution task. The overall results show that children with ASD can distinguish moral from conventional transgressions irrespective of the status of their ToM skills (Fadda et al., 2016; Grant et al., 2005; Margoni and Surian, 2016; Moran et al., 2011; James and Blair, 1996; Kretschmer et al., 2014; Lecciso et al., 2008; Leslie et al., 2006; Patil et al., 2016; Shulman et al., 2012; Zalla et al., 2011), but they tend to experience subtle differences on advanced moral tasks requiring the interpretation of others’ intentions (Dempsey et al., 2020b; Mathersul et al., 2013; Senland and Higgins-D’Alessandro, 2016).

Narrative comprehension studies in ASD (e.g., Losh and Gordon, 2014; Mäkinen, 2015; Westerveld et al., 2021) often ask children to make explicit mental state attributions without investigating their moral justification of the story characters’ decision-making and their ability to form moral linkages between the characters’ actions and the outcomes of the story. Perspective-taking that relies on morally salient narrative contexts has not been sufficiently investigated in autistic individuals, which prevents us from understanding whether differences in moral thinking between autistic and TD children are a potential additional source of variation in the mental state attribution skills of autistic individuals.

The current study aimed to assess cognitive and affective ToM, as well as moral reasoning skills in autistic children through Aesop’s fable comprehension. In considering the narrative design of the current study, Aesop’s fables seemed like an appropriate venue to assess autistic children’s mental state attribution skills. Fables may be regarded as didactic stories that transform a wise general thought, known as the moral or life lesson, into a brief and simple plot (Konstantakos, 2025). Aesop’s fables usually describe events enacted by animals that talk to each other comprehensibly in human speech. The use of non-humans makes them appealing due to children’s natural attraction to stories about animals (Hall, 2016; Korhonen, 2019), but most importantly, because they implicitly introduce children to the nature of human thoughts, intentions and desires. Specifically, Aesop’s fables are narratives which operate on two levels; on the surface, the storyline involves two or three animal characters that go on adventures; beneath this surface, however, lies a deeper meaning. These stories often explore the relationships between characters with different levels of physical strength or intelligence. One character usually holds a mistaken view of reality, which leads to conflict or misunderstanding (Patterson, 1991). To grasp these relationships, children need to be able to understand the characters’ conflicting perspectives of the same situation and, more importantly, judge the morality of the strategies the protagonists resort to, in order to reach their goals. These strategies (e.g., trickery, flattery, trading favors) can sometimes clash with the idea of a fair and just world. This particular aspect gives Aesop’s fables a distinctive moral resonance that is not always as prominently featured in other narrative tools, thus, making them especially effective for introducing complex ethical concepts to young audiences (Jose et al., 2005). Aesop’s fables have been extensively used as measures of inferential skills in preschool-aged (Pelletier and Beatty, 2015; Zhang et al., 2025) and older children (Peristeri et al., 2026b), as their content, style and genre have been considered appropriate to provide rich language and literacy learning opportunities to children of this age-range cross-culturally. Especially, in the Greek educational context, wherein the current study has been conducted, the use of fables has been established in the Greek National Curriculum for kindergarten through the use of the ‘Neohellenic Language and Literature’ textbook (Magoula et al., 2022) for preschool and early primary school education. Aesop’s fables have been a popular method of teaching literacy skills in Greek kindergartens and primary schools. The current study will capitalize on Aesop’s fables’ moral dimension to investigate moral judgment in children on the spectrum, since the function of most of the fables is to direct the child toward a specific moral interpretation of the story characters’ actions, and a reflection about the moral impact of the outcomes of the protagonists’ actions.

Beyond the moral force characterizing Aesop’s fables, the latter seem to carry a number of extra advantages. First, they have a simple plotline that comprises few episodes and two-to-three protagonists, thus, they are not characterized by high cognitive complexity. Next, Aesop’s fables are representative of genuine social interactions as the animal protagonists get themselves into similar conflict situations or dilemmas as humans do, e.g., flattering someone to make him/her feel important, aspiring to things which are not naturally yours, or considering small creatures to be less powerful than big ones. The fables’ connection to social situations and interactions seems to add to their ecological validity as tools assessing children’s mentalizing and moral reasoning skills.

The current study has employed two Aesop’s fables, namely, ‘The fox and the crow’ and ‘The mouse and the lion’, which were differentiated by variations in their linguistic complexity, including vocabulary and syntax (see Methodology). Furthermore, the two stories critically differed in their frequency of use of decontextualized language, i.e., expressions that anchor events in the past. Specifically, the time of occurrence of the story in ‘The lion and the mouse’ shifts back and forth throughout, when the story refers to events in the past, and past and present events are interlaced as the two characters dialog back and forth. On the other hand, the use of decontextualized language in ‘The fox and the crow’ is minimal, since focus is predominantly placed in events in the present. According to previous research (Rudling et al., 2023; Uccelli et al., 2019), the use of decontextualized language is an early emerging pre-pragmatic ability, potentially suitable for studies of pragmatic skills in ASD due to prominent difficulties that autistic children face in the specific language subdomain (Botting, 2002; Norbury et al., 2014; Peristeri et al., 2017, 2020, 2024a; Themistocleous et al., 2024).

To determine whether differences in narrative comprehension between the autistic and TD children in the current study stemmed from potential delays in the autistic children’s language abilities or from a deviant developmental pattern, we included two TD control groups: one matched for age and the other for language. By matching the autistic and TD children on language, we ensured that two groups would have comparable lexical and morphosyntactic skills (e.g., Parish-Morris et al., 2007; Venker, 2019; Andreou et al., 2025a), which would in turn allow us to isolate differences between the groups in ToM and moral reasoning.

We have asked the following two research questions:

Research question (i): *Will autistic children’s affective and cognitive ToM attribution and moral reasoning skills in Aesop’s fables differ from TD age-matched or/and language-matched children?*

Hypothesis: Based on previous narrative studies (e.g., Losh and Gordon, 2014; Mäkinen, 2015; Westerveld et al., 2021) that have found considerable ToM difficulties in autistic children as compared to TD peers, we expected that the autistic children in the current study would score lower than the age-matched TD group in attributing affective and cognitive ToM states to the protagonists of the fables. The same pattern was expected for moral reasoning, which was assessed through a question asking them to tell/verbalize the life lesson, i.e., the moral, of each fable. It remains an open question whether ToM attribution and moral judgment skills in ASD would be affected by the children’s language abilities; if ToM and moral judgment are tied to structural language abilities, we expected that the autistic and TD language-matched group would perform at similar levels. In case of differences between the autistic and the TD language-matched group, this would suggest deficits in mentalizing and moralizing for the autistic group that go beyond the structural language level. The current study is the first to investigate the effect of language ability on autistic children’s ToM and moral judgment skills within the same task.

Research question (ii): *Will autistic children’s affective and cognitive ToM attribution and moral reasoning skills differ across the two fables?*

Hypothesis: Based on previous findings that processing decontextualized language is challenging for autistic children (e.g., Botting, 2002; Norbury et al., 2014), along with the fact the ‘The mouse and the lion’ fable employs more decontextualized language than the ‘The fox and the crow’, we expected that the autistic group would score lower in the ‘The mouse and the lion’ fable.

## 2 Materials and methods

### 2.1 Participants

The study included three experimental groups, specifically, one including 25 monolingual Greek-speaking children with ASD (18 boys, mean age: 8 years, 9 months), and two control groups, namely, 25 TD children who were matched to the autistic group on chronological age (18 boys, mean age: 8 years, 6 months), and 25 TD children (18 boys, mean age, 6 years, 2 months), who were chosen to match the autistic group on language measured via a standardized expressive single word vocabulary test (Vogindroukas et al., 2009; Renfrew, 1997b) and a sentence repetition task [Diagnostic Verbal IQ Test (DVIQ); Stavrakaki and Tsimpli, 2000]. The sentence test is not standardized in Greek. This test consists of 10 items involving a variety of morphosyntactic structures in Greek, such as embedded relatives, adjuncts, co-ordinate and passive sentences. Participants were asked to listen to the 10 pre-recorded sentences from the test spoken by the first author, who is a native speaker of Greek, to ensure that all participants hear the sentences in the same way. After listening to each sentence, children were asked to repeat the sentence as close to the original as possible. Each child received a score of 3 in case no errors were committed, 2 points in case of a single error, 1 point in case of two errors, and 0 points in case three or more errors were committed. Table 1 shows the children’s scores on the two language screening tests that were used to match autistic and TD children on their language ability. We should note that, in the sentence repetition task, and in spite of the errors made on agreement or/and tense inflections in the sentences or the lexical paraphasias made, the autistic children could repeat complex sentences, such as passives and relative clauses, with slight changes in word order, which nevertheless did not distort the meaning of the sentences. This performance was a prerequisite for administering the narrative production task, in which children were required to produce continuous stretches of discourse.

**Table 1:** Participants’ mean expressive vocabulary and sentence repetition scores (along with SD and ranges).

Group	Expressive vocabulary (max. 50)	Sentence repetition (max. 30)
ASD (*n* = 25)	22.1 (5.2) 12–42	18.9 (6.2) 5–30
TD1 (*n* = 25)	47.5 (1.8) 44–50	28.1 (2.1) 22–30
TD2 (*n* = 25)	24.0 (5.5) 17–34	18.6 (4.4) 6–28

ASD, autistic children; TD1, age-matched typically-developing children; TD2, language-matched typically-developing children; SD, standard deviation; max., maximum score.

Regarding the sample size, Pelletier and Beatty’s (2015) reading comprehension study with Aesop’s fables included 21 TD children per age group, and had a power of 70.9% to detect group differences at the *p* < 0.05 level. Power analyses in the current study, using the present sample size (i.e., 25 participants per group), indicated an actual power of 77.3% to detect group differences at the *p* < 0.05 level in the same conditions of the bridging inferencing task.

All autistic children had a Wechsler Performance IQ over 75 [PIQ; Wechsler Intelligence Scales for Children-Third Edition (WISC-III); Wechsler, 1991; Greek version by Georgas et al., 1997], which is the critical threshold indicating intellectual potential within the normal range and high-functioning autism (e.g., Peristeri and Andreou, 2024; Peristeri et al., 2022; Riccioni et al., 2021). According to parental and teacher reports, autistic children demonstrated a good level of language proficiency, thus they were all highly-verbal, and could also provide information about events beyond the immediate context. For the non-autistic groups, parents confirmed that their children did not have an autism diagnosis or any other developmental disorder. The age-matched TD children’s (henceforth, TD1 group) PIQ was measured through WISC-III (Wechsler, 1991; Greek version by Georgas et al., 1997), while the younger, language-matched TD children’s (henceforth, TD2 group) PIQ was assessed through the Wechsler Preschool and Primary Scales of Intelligence-Third Edition (WPPSI-III; Wechsler, 2002; Greek version by Sideridis and Antoniou, 2015). Table 2 shows the children’s ages and their scores on nonverbal intelligence (PIQ; WISC-III; Wechsler, 1991; Greek version by Georgas et al., 1997, for the autistic and the TD age-matched group; WPPSI-III; Wechsler, 2002; Greek version by Sideridis and Antoniou, 2015, for the TD language-matched group). A series of one-way ANOVA analyses showed that there was a significant Group effect in age, *F*(2, 72) = 74.100, *p* < 0.001, *η*^2^ = 0.68). Post-hoc tests evinced that there was no significant difference between autistic and TD1 children (*p* = 0.984); however, both groups were significantly older than the TD2 group (*p* < 0.001 for both differences). There was no significant Group effect in PIQ scores, F(2, 72) = 1.758, *p* = 0.180, η^2^ = 0.04).

**Table 2:** Participants’ mean ages and PIQ scores (along with SD and ranges).

Group	Age (years; months)	PIQ
ASD (*n* = 25)	8;9 (1.4) 7;1–11;1	85.2 (7.0) 75–92
TD1 (*n* = 25)	8;6 (1.5) 7–11	87.6 (4.3) 77–94
TD2 (*n* = 25)	6;2 (0.5) 5;1–6;9	88.5 (4.1)76–93

PIQ, Performance IQ; ASD, autistic children; TD1, age-matched typically-developing children; TD2, language-matched typically-developing children; SD, standard deviation.

Participants were recruited from the regions of northern and central Greece, respectively. Referrals for the autistic group were made by Centers for Interdisciplinary Evaluation, Counseling, and Support, which are official state centers in Greece responsible for assessing and supporting the educational needs of preschool and school-aged students, including those with disabilities or special educational needs. All autistic children had received a formal clinical diagnosis of ASD from a child psychiatrist or developmental specialist, based on the diagnostic criteria outlined in the DSM-5 [American Psychiatric Association (APA), 2013] and ICD-10 (World Health Organization, 1993). We should mention that the autistic children that have participated in the current study had just received their first diagnosis from the Centers for Interdisciplinary Evaluation, Counseling, and Support, which was the primary recruitment site of the study. As such, none of the autistic children had received speech-language pathology prior their participation in the study. As the centers in the specific geographical region serve a diverse and economically disadvantaged population, children tend to receive a diagnosis rather late with more than half of the cases of autism (57%) being diagnosed in the 8–10 years age group (Thomaidis et al., 2020). Many factors likely contribute to the late age of diagnosis, including limited access to the diagnostic services throughout central Greece, especially for the families living in mountainous areas, long wait times for appointments with the average wait time being more than a year, and the caregivers’ low acceptance of autism risk.

All children attended mainstream classes in public schools. Study procedures were approved by the Aristotle University of Thessaloniki Institutional review board (IRB protocol number: 39928/20.02.2024).

### 2.2 Narrative comprehension task

The current study has employed two Aesop’s fables with two main characters each: ‘The fox and the crow’ and ‘The lion and the mouse’. Both fables were retrieved from the textbook of ‘Modern Greek Language and Literature’, which has been developed by the Greek Institute of Educational Policy (Magoula et al., 2022; see Supplementary materials). According to the parents’ reports, none of the children that participated in the current study was familiarized with any of the two fables.

What follows is short descriptions of the content of each fable.

*The fox and the crow*. This fable describes a crow holding a piece of cheese and a hungry fox that speaks cleverly and carries out a deception in order to steal the crow’s piece of cheese. In order to achieve her desire, the content of the fox’s speech becomes sinister as the fox stresses that the crow is beautiful enough to be a king, but it is a great pity that she has not yet had the chance to listen to its wonderful voice. The crow opens its mouth to sing, thus dropping the cheese which lands into the fox’s mouth. Then, the fox begins the insult saying that of course the crow does not have a voice, but it does not have a brain either. The moral of the fable is that the perceived deficiency exploited by the fox is the crow’s lack of intelligence, not a lack of voice.

*The lion and the mouse.* The plot follows a mouse that disturbs a lion from rest in its nest, thus, being in danger of being eaten by the lion. The lion makes a life-changing decision to spare the mouse’s life. No sooner after the mouse is released, two poachers set up a snare that eventually traps the mighty lion. The mouse remembers its debt, eats its way through the twine and releases the lion from the poachers’ trap. At the end of the fable, the two creatures become true forever best friends. The moral of the fable is inspired by seeing the majestic king of the jungle being saved by the determination and hard work of a humble creature, and offers a message of how the meek can trump the mighty.

To identify possible structural language differences between the two fables, their lexical and syntactic features were automatically computed through Open Brain AI (Themistocleous, 2024), which is a comprehensive computational platform that provides flexible Artificial Intelligence language analysis solutions for written language in Greek. Measures provide the total number of words and sentences in the text; the text’s count of content and function words (coordinating conjunctions, determiners, particles, pronouns, and subordinating conjunctions); type-token ratio (TTR), i.e., the ratio of unique words (types) to the total number of words (tokens) in the text, multiplied by 100 to convert it to a percentage; the total number of adjectives/clauses/simple clauses/coordinate clauses/subordinate clauses; and the mean sentence length, computed as the average number of words per sentence in the text. As one can see in Table 3, ‘The lion and the mouse’ story had higher lexical diversity and syntactic complexity scores than ‘The fox and the crow’, which is evinced in the first story’s higher TTR, higher number of subordinate clauses and lower number of simple clauses. These metrics reflect the structural language complexity of the fables, rather than their use of decontextualized language and lexical viewpoint markers, that refer to past events and story characters’ affective and cognitive mental states, respectively.

**Table 3:** Lexical and syntactic measures for ‘The fox and the crow’ and ‘The lion and the mouse’ fable.

Fable	Total no of words (content/function)	TTR (%)	No of clauses (raw scores)	No of simple clauses (raw scores)	No of coordinate clauses (raw scores)	No of subordinate clauses (raw scores)	Mean sentence length (average number of words)
The fox and the crow	288 (248/40)	45.9	38	6	10	22	12.08
The lion and the mouse	304 (268/36)	58.6	38	1	9	28	12.16

no, number; TTR, type-token ratio.

*Fable comprehension procedure*. After listening to each pre-recorded fable by the same female speaker, children were asked six questions. Three of them assessed their ability to attribute emotions to the two characters in each fable, thus, they assessed children’s affective ToM skills (see questions 1–3). Two questions tested children’s ability to attribute cognitive mental states to the characters of each fable (see questions 4 & 5). The sixth question asked them to tell the life lesson, i.e., the moral, of the fable (see question 6). The moral was not explicitly mentioned in either of the two fables the children listened to. The questions along with their scoring were adapted from Pelletier and Beatty’s (2015) reading comprehension study with TD children using Aesop’s fables. Children’s answers were both recorded and noted down by the experimenter. Each answer was coded as correct/incorrect (0 or 1; see Pelletier and Beatty, 2015), so the total accuracy score for each fable was 6. No child participant has asked to listen to the fables twice. Children’s answers were first coded by the first author, and were then double-coded for reliability purposes by the final author. Inter-rater agreement reached 97%

(1) How did the fox feel when she saw the cheese in the crow’s mouth? (‘The fox and the crow’) / How did the mouse feel when the lion caught it? (‘The lion and the mouse’)
(2) How did the crow feel when the piece of cheese fell off its mouth? (‘The fox and the crow’) / How did the lion feel when the poachers trapped it? (‘The lion and the mouse’)
(3) How did the fox feel when she took the cheese from the crow? (‘The fox and the crow’) / How did the mouse feel when it freed up the lion? (‘The lion and the mouse’)
(4) What did the fox think about the crow when she saw it on the tree? (‘The fox and the crow’) / What did the lion think about the mouse when the mouse got in his nest? (‘The lion and the mouse’)
(5) What did the crow think about its voice when singing? (‘The fox and the crow’) / What did the mouse think about the lion when it was trapped? (‘The lion and the mouse’)
(6) What can one learn from the whole story? (both fables)

The tasks were administered in three sessions that took place on different days at the children’s home or school. Session 1 included the non-verbal intelligence measurements; as already mentioned the PIQ of the autistic and the older TD age-matched group was measured via the Greek version of WISC-III (Wechsler, 1991; Greek version by Georgas et al., 1997), while the younger TD age-matched group was administered the WPPSI-III (Wechsler, 2002; Greek version by Sideridis and Antoniou, 2015). WISC tools were administered by a licensed clinical psychologist. Session 2 included the expressive vocabulary test and the first fable comprehension task, and session 3 included the sentence repetition test and the second fable. To avoid stimulus order effects, the order of the two narrative comprehension sessions was counterbalanced, i.e., 13 of the children in each group (autistic, age-matched TD, language-matched TD) received the tasks of session 1 first, and the rest received the tasks of session 2 first.

## 3 Analyses plan

We first provide descriptive values of the three groups’ (ASD, age-matched TD, language-matched TD) performance means in the two language screening tests, specifically, expressive vocabulary and sentence repetition. We next ran one-way ANOVA analyses to detect group differences in the above measures. To test both research hypotheses, i.e., investigate whether the three groups would differ in narrative comprehension, we ran two-way ANOVA analyses using Group (ASD, TD1, TD2) and the Fable (Fox/Lion) as independent factors, and the three narrative comprehension measures (affective ToM, cognitive ToM, moral comprehension) as the dependent variables. Furthermore, an *a priori* power analysis was conducted using G*Power (Faul et al., 2007) to test the difference between three independent group means using analysis of variance, a large effect size (d = 0.40), and an alpha of 0.05. The result showed that a total sample of *n* = 80 is acceptable for the study to achieve a power of 0.80. Our total sample consists of 75 children.

## 4 Results

One-way ANOVA analyses showed a significant Group effect in expressive vocabulary, *F*(2, 72) = 93.590, *p* < 0.001, η^2^ = 0.87). Post-hoc tests evinced that there was no significant difference between autistic and TD2 children (*p* = 0.123), however, both groups scored significantly lower in the expressive vocabulary test than the TD1 group (*p* < 0.001, η^2^ = 0.86 for the difference between autistic and TD1 children; *p* < 0.001, η^2^ = 0.83 for the difference between TD2 and TD1 children). In sentence repetition, we found a significant Group effect, *F*(2, 72) = 35.769, *p* < 0.001, η^2^ = 0.48). Post-hoc tests evinced there was no significant difference between autistic and TD2 children (*p* = 0.974), however, both groups scored significantly lower in sentence repetition than the TD1 group (*p* < 0.001, η^2^ = 0.78 for the difference between autistic and TD1 children; *p* < 0.001, η^2^ = 0.75 for the difference between TD2 and TD1 children).

Table 4 presents the three groups’ mean scores in each of the categories comprising narrative comprehension, namely, affective ToM, cognitive ToM, and moral comprehension. Total comprehension accuracy is also included in Table 4. Scores are reported separately for each fable (‘The fox and the crow’, ‘The Lion and the mouse’).

**Table 4:** Groups’ mean affective ToM, cognitive ToM, and moral comprehension scores (SD within the parentheses) in each Aesop’s fable.

Group	Fable	Affective ToM (max. 3)	Cognitive ToM (max. 2)	Moral (max. 1)	Total (max. 6)
ASD (*n* = 25)	Fox	2.1 (0.5)	1.6 (0.6)	0.6 (0.5)	4.2 (1.1)
TD1 (*n* = 25)		2.6 (0.5)	1.9 (0.4)	0.9 (0.2)	5.4 (0.7)
TD2 (*n* = 25)		2.2 (0.8)	0.9 (0.6)	0.3 (0.4)	3.3 (1.4)
ASD (*n* = 25)	Lion	2.0 (0.7)	0.7 (0.8)	0.1 (0.4)	2.8 (1.6)
TD1 (*n* = 25)		2.8 (0.5)	1.9 (0.4)	0.7 (0.4)	5.4 (0.9)
TD2 (*n* = 25)		2.4 (1.1)	1.2 (0.4)	0.6 (0.5)	4.0 (1.7)

ASD, autistic children; TD1, typically-developing age-matched children; TD2, typically-developing language-matched children; SD, standard deviation; ToM, theory of mind.

The two-way ANOVA analyses were run separately for each category, namely, affective ToM, cognitive ToM, and moral comprehension. Analyses were also run on the total comprehension accuracy scores collapsing across categories.

The analysis on affective ToM comprehension scores revealed a significant effect for Group, *F*(2, 72) = 4.772, *p* = 0.012, η^2^ = 0.11, which stemmed from the fact that the autistic group scored significantly lower than the TD1 group (*p* = 0.011); the differences between the autistic and the TD2 group (*p* = 0.337), and between the TD2 and the TD1 group (*p* = 0.09) were not found to be significant. The Fable effect, *F*(1, 72) = 0.708, *p* = 0.404, η^2^ = 0.01, and the two-way interaction between Group and Fable on affective ToM comprehension accuracy, F(1, 72) = 0.878, *p* = 0.421, η^2^ = 0.01, were not found to be significant. Paired t-tests showed that affective ToM comprehension did not differ across the two fables for either group (t(24) = 0.768, *p* = 450 for the ASD; t(24) = 0.681, *p* = 0.503 for the TD1; t(24) = 1.163, *p* = 0.256 for the TD2 group).

The analyses on cognitive ToM comprehension scores revealed a significant Group effect, *F*(2, 72) = 20.185, *p* < 0.001, η^2^ = 0.25; post-hoc analyses revealed that the TD1 group exhibited significantly higher accuracy in cognitive ToM comprehension than both the autistic and the TD2 group (*p* < 0.001, η^2^ = 0.23 for the difference between TD1 and autistic children; p < 0.001, η^2^ = 0.25 for the difference between TD1 and TD2 children), while the difference between the autistic and TD2 children was not significant (*p* = 0.716). The Fable effect was found to be significant, *F*(1, 72) = 3.988, *p* = 0.05, η^2^ = 0.02, and stemmed from the fact that cognitive ToM comprehension in the Fox fable was significantly higher than the Lion fable. The two-way interaction between Group and Fable was highly significant, F(2, 72) = 11.051, *p* < 0.001, η^2^ = 0.11, and it stemmed from the performance of the autistic group; specifically, autistic children’s cognitive ToM performance in the Fox fable was considerably higher than the Lion, t(24) = 4.529, *p* < 0.001. The difference between the two fables in terms of cognitive ToM scores was not significant for either the TD1, t(24) = 0.01, *p* = 1.0, or the TD2 group, t(24) = 1.441, *p* = 0.505. In moral comprehension, there was a significant Group effect, F(2, 72) = 12.002, *p* < 0.001, η^2^ = 0.10; post-hoc analyses revealed that both the autistic and the TD2 group had significantly lower scores in moral comprehension than the TD1 group (*p* < 0.001, η^2^ = 0.18 for the difference between TD1 and autistic children; *p* < 0.001, η^2^ = 0.17 for the difference between TD1 and TD2 children), while the difference between autistic and TD2 children was not significant (*p* = 0.820). The Fable effect was not found to be significant either, F(1, 72) = 3.244, *p* = 0.077, η^2^ = 0.015. However, there was a significant two-way interaction between Group and Fable, F(2, 72) = 11.585, *p* < 0.001, η^2^ = 0.10. Paired t-tests between performance across the two fables in each group showed that the moral comprehension score in the Fox was significantly higher than the Lion for the autistic children only, t(24) = 4.011, *p* = 0.002, while the reverse pattern was observed for the TD2 group, i.e., moral scores were higher in the Lion (vs. Fox) fable, t(24) = 2.009, *p* = 0.05. No difference between the two fables was observed for the TD1 group, t(24) = 1.783, *p* = 0.400.

Finally, the analyses on the total comprehension accuracy scores revealed a significant Group effect, F(2, 72) = 16.138, *p* < 0.001, η^2^ = 0.28; post-hoc analyses revealed that both the autistic and the TD2 group had significantly lower scores than the TD1 group (*p* < 0.001, η^2^ = 0.26 for the difference between TD1 and autistic children; *p* < 0.001, η^2^ = 0.23 for the difference between TD1 and TD2 children), while the difference between autistic and TD2 children was not significant (*p* = 0.623). The Fable effect was not significant, F(1, 72) = 1.991, *p* = 0.164, η^2^ = 0.01. There was a significant two-way interaction between Group and Fable, F(2, 72) = 14.397, *p* < 0.001, η^2^ = 0.08. Paired t-tests between the two fables in each group showed that the total comprehension score in the Fox was significantly higher than the Lion for the autistic children only, t(24) = 4.888, *p* < 0.001, while no difference was observed for either the TD1, t(24) = 0.175, *p* = 1.00, or the TD2 group, t(24) = 2.619, *p* = 0.08.

## 5 Discussion

In the current study, we report findings on the ToM and moral comprehension skills of school-aged autistic children from Aesop’s fables, a newly used measure of ToM and moral judgment abilities in ASD. Aesop’s fables were employed to provide a sensitive ToM measure, characterized by a simple plot, and capable of picking up on variability in autistic children’s affective and cognitive ToM understanding. Notably, besides answering questions that assessed the attribution of affective and cognitive mental states to the fable protagonists, the children were also assessed on their moral reasoning skills by providing the life lesson, or else the moral, of each fable. We found that the autistic children had difficulty attributing both affective and cognitive mental states to the story protagonists as compared to their age-matched peers, and that cognitive ToM skills worsened with higher fable complexity. Also, the autistic children exhibited considerable weaknesses in verbalizing the moral of the fables, however, this weakness appeared to be dependent on the language complexity and the story structure of the fables.

The first research question of the current study aimed to investigate whether the autistic children would differ from age- and language-matched TD children in their affective and cognitive ToM attribution skills, as well as in their ability to understand the moral of each fable. According to the results, the autistic children faced difficulty with attributing both emotions and cognitive states to the fable protagonists, since they scored significantly lower than their age-matched TD peers in both ToM categories, but similarly to the TD language-matched group. The results are similar to previous research (Peristeri et al., 2017; Peristeri et al., 2024b; Sah and Torng, 2019; Tager-Flusberg and Sullivan, 1995), supporting that autistic children lag behind TD peers because of language limitations in vocabulary and syntax, given the close interconnections between language, executive functioning and ToM development in ASD (Andreou et al., 2026b). The particular studies have shown that autistic children with lower lexical and morphosyntactic skills as compared to their TD peers tend to show greater difficulty with attributing the appropriate mental state terms to story characters in narrative production. Interestingly, the younger TD children in the current study tended to score lower than the older TD group in cognitive ToM attribution, while the two TD groups did not differ in their affective ToM attribution performances, further suggesting that cognitive and affective ToM development may follow different developmental trajectories (Gabriel et al., 2021; Sebastian et al., 2012). We should note, however, that this pattern of results should be interpreted through the lens of the specific methodological tool, and more specifically, Aesop’s fables wherein the protagonists’ emotions, beliefs and moral intentions were inextricably interwoven. As such, the specific findings about the autistic children’s affective and cognitive ToM skills should be regarded as layered evidence possibly affected by cognitive and moral reasoning abilities, rather than being attributed to a single impairment in children’s ToM mechanism.

The study’s second research question was to investigate the effect of the fables’ decontextualized language on autistic (and TD) children’s comprehension skills. We found that the autistic group’s cognitive ToM comprehension performance was more negatively affected in the Lion (vs. Fox) fable. This finding was expected, since the Lion fable included several events removed from the here and now, thus, employed to a great extent decontextualized language structures, including elaborate syntax, to increase the specificity of causal or temporal relations between the past events that led up to the ending of the story (Curenton et al., 2008; Reese, 1995; Rowe, 2013; Uccelli et al., 2019). Based on evidence that the use of cognitive mental state terms is one of the main components of decontextualized language (Greenhalgh and Strong, 2001; Pellegrini, 1985), and that the Lion fable involved more instances of decontextualized language than the Fox, we hypothesize that the processing of cognitive viewpoints was possibly more effortful for the autistic group for the particular fable. On the other hand, the contextualized frame of the Fox story seemed to facilitate children’s ability to make sense of the cognitive inner world of the story characters. Our findings about the challenging nature of decontextualized language processing in ASD seem to agree with Su et al. (2024) longitudinal study that has found that the growth of decontextualized language (including reference to cognitive mental states in past events) in young autistic children follows a protracted developmental trajectory relative to other language measurements, including mean length of utterance and word combinations, which exhibited a rather steady growth over a year’s span. Of course, more research is warranted to determine the effects of decontextualized language on ToM attribution skills in ASD.

Regarding children’s performance in the questions targeting moral comprehension, autistic children and their language-matched peers exhibited significantly lower accuracy rates than the older TD group. This pattern suggests an impairment in the autistic group’s moral reasoning skills, and adds to previous research showing that autistic individuals experience subtle difficulties on moral tasks requiring the interpretation of others’ intentions (Mathersul et al., 2013), or have less advanced moral reasoning skills (Senland and Higgins-D’Alessandro, 2016). Though providing the moral of the fables involves inferential skills, the fact that autistic children’s performance in the specific question was significantly lower than their TD peers across fables, suggests that moral reasoning in the autistic group probably tapped into distinct cognitive resources as compared to the rest of the questions. Interestingly, the results revealed a divergent pattern of performance between the autistic and language-matched TD children across the two fables, since the autistic group was more competent at verbalizing the moral of the Fox (vs. Lion) fable, while the opposite pattern was found for the language-matched TD group. This dissociation could not have stemmed from differences in the language demands of the two fables, since the two groups were language-matched. A qualitative inspection of the autistic group’s answers to the questions targeting the moral of the two fables shows that the autistic children could more easily grasp the moral lesson of the Fox fable, specifically that foolishness may be harmful to one’s interests, rather than the life lesson in the Lion fable, that being physically weak is not an obstacle to making achievements. The majority of the autistic children’s answers to the critical question in the Lion fable were either elusive (e.g., “People should be tender to animals,” “Lions are endangered animals”), or tended to place emphasis on specific details of the story (e.g., “We need to protect the forest and its animals,” or “We need to be careful when we walk in the forest, in case we fall in a hidden trap”), without taking into consideration the two animals’ disparate physical power. We speculate that the moral in the Lion fable required more advanced reasoning skills to understand that physical weakness can be compensated by intellectual strength. This assumption receives partial support from the performance of the older TD group whose performance in the moral comprehension question in the Lion fable was rather moderate (i.e., 70%). We may thus hypothesize that the advanced moral reasoning demands in the Lion fable made its life lesson more difficult to grasp or/and verbalize.

Another possible scenario for the finding that the autistic children were more efficient at understanding the moral in the Fox (vs. Lion) fable is because the former fable was tied to a clear and concrete goal-attempt-outcome sequence, also known as story grammar (Stein and Glenn, 1979; i.e., the fox was hungry, it deceived the crow and got the cheese). The clear story grammar of the Fox story enabled the autistic children to moralize without burdening their inferencing about the story characters’ intentions. On the other hand, the moral in the Lion fable relied strongly on multi-layered social knowledge, including concepts such as physical power inequality, altruism and intellectual inner strength, as well as the integration of non-literal and higher-order meanings, which are often deficient in autistic individuals (Andreou et al., 2025b; Gates et al., 2023; Keifer et al., 2021; Livingston et al., 2019; Peristeri et al., 2014). Meanwhile, language-matched TD children faced less difficulty grasping the moral in the Lion fable, while they were poorer at understanding the moral of the Fox fable, likely because story grammar skills at the age of five are still under development (e.g., Chen et al., 2011; Filiatrault-Veilleux et al., 2015; Shunhua and Tianlong, 2023) and, thus, could not successfully scaffold moral reasoning in the Fox fable.

## 6 Limitations

Several limitations in the current study need to be mentioned. First, we probed the comprehension of morals across Aesop’s fables with a single question per fable, which may have limited the sensitivity of the measure and our potentials to detect individual differences in the groups. Also, differences in the number of questions or scoring opportunities may have affected score comparability and could have influenced the observed group differences. Furthermore, Aesop’s fables include a diversity of mental processes, including intentions, beliefs, moral transgressions and emotions that are hard to tease apart in a meaningful way. The administration of separate ToM tests and their relation to moral judgment in the fables could have provided a deeper understanding of the relations between ToM and moral judgment skills in ASD. Second, the use of more fables matched on narrative structure and linguistic complexity could have captured a wider range of children’s performance in comprehending the morals, which would in turn have allowed a deeper grasp of the autistic children’s moral judgment skills. Alternatively, linguistic complexity in the fables could have been operationalized in terms of lexical frequency, use of epistemic language and mental state vocabulary, besides TTR and syntactic complexity that have been measured in the current study. Also, language matching in the current study could have been operationalized in terms of additional language measures, such as receptive vocabulary or a syntactic comprehension task. Finally, the present study only included autistic children with PIQs in the average range, which reduces the generalizability of the findings to children across the spectrum. Future research designs should assess autistic children with different levels of functionality to gain insight into narrative comprehension nuances of the entire spectrum.

## 7 Conclusion

The current narrative comprehension study adds to previous findings on ToM attribution skills in autistic children through a narrative genre of wisdom literature, namely Aesop’s fables. The findings also show that Aesop’s fables offer a promising venue as a window into the moral judgment skills of autistic children, which is ecological-friendly and sensitive to children’s individual differences in language and ToM abilities. These benefits may translate into gaining more reliable insights in the ToM attribution and moral reasoning skills in ASD. There is still much progress to be made in narrative comprehension assessment in autistic children, and we hope that the use of Aesop’s fables as a narrative assessment tool will contribute to this endeavor.

## Data Availability

Data will be made available on request to the corresponding author.
