# Supplementary material for: Narrative discourse comprehension in Greek-speaking children with autism spectrum disorder: evidence from Aesop’s fables
Source: Front Psychol. 2026 Sep 24;17:1877679. doi: 10.3389/fpsyg.2026.1877679 (PMC13645775; doi:10.3389/fpsyg.2026.1877679)
Supplement: Supplementary file 1 [file Supplementary_file_1.docx]

Supplementary Material

# 1  “The fox and the crow”

Once upon a time, there was a fancy crow who thought it was smart and good-looking. One day, the crow found a piece of cheese and sat on the branch of an olive tree to eat it in peace. At that moment, a cunning and hungry fox happened to be passing under the tree. As soon as the fox saw the crow, it drooled over the piece of cheese. Since the fox could not climb up the tree to steal the cheese from the crow, it started thinking how it could trick it and take the cheese. After thinking carefully, the fox said to the crow: “What a beautiful bird you are! Your plumage is shiny black! Your legs are thin and beautiful! And your nails look like pearls. Your eyes are the most beautiful and intelligent in the world, and your beak is the envy of all birds!’. The crow, who was dumb, began to strut on the branch with pride and joy. The fox continued saying: ‘With so much beauty and intelligence that you have, you should be the king of all birds. I would like to hear your voice so much! I am sure that it will be sweeter than the nightingale’. Having heard all that, the crow got very happy and opened its beak to sing. But as soon as it opened its beak, the cheese fell off its mouth, and the fox grabbed it and ate it. Then the fox turned to the crow and said: “You fool! Stop your awful singing. If you had any sense, you would have eaten the cheese and I would still be hungry. But I made fun of you and ate your cheese. Next time, be more careful of those who say kind words to you’.

# 2 ‘The lion and the mouse’

## Once upon a time, a huge lion was sleeping in the shade of an old tree when a mouse climbed onto its front paws and mane. The little mouse played carefree with the long hair of the lion’s mane until at some point the huge beast opened its eyes and grabbed the mouse. As the lion was about to eat it, the frightened mouse began to beg the lion to have mercy on it: ‘I am so sorry, my dear lion. I didn’t mean to bother you. Forgive me and I promise that, if you set me free, I will repay you for your kindness and help you if you ever need me’. The lion burst into laughter and said: ‘How could you even think that that I could ever need your help, little creature? However, because you made me laugh, I will spare your life. Go ahead, you can leave’. The next day, the lion was caught in a trap set by hunters who wanted to catch the lion alive. The lion was desperate when the little mouse happened to pass by. It heard the lion’s roars and found where it was trapped. The little mouse recognized the lion who had saved its life and without a second thought it started biting the ropes that had tied the lion with its sharp teeth, until it cut them and set the lion free. Then the mouse said to the lion: ‘A few days ago, you laughed at me because you thought that I could never do anything for you. But now you see that even though we, mice, are small creatures, we know how to keep our promises and sometimes we can help big animals’. The lion and the mouse swore to remain friends
